# Younger, drunk, and fast: Paradoxical rapid reaction time in hazardous drinkers

**DOI:** 10.1177/02698811231177216

**Published:** 2023-05-24

**Authors:** Anna Powell, Harry Sumnall, Catharine Montgomery

**Affiliations:** 1 School of Psychology, Liverpool John Moores University, Liverpool, UK; 2 Liverpool Centre for Alcohol Research, Liverpool, UK; 3 Public Health Institute, Liverpool John Moores University, Liverpool, UK

**Keywords:** Cognitive function, vibrotactile perception, executive function; alcohol, hazardous alcohol use

## Abstract

**Background:**

Research consistently links hazardous alcohol use with reduced cognitive function but is less consistent with regard to processing speed, which underpins many cognitive functions. Using vibrotactile perception to assess cognitive function may have benefits over other sensory stimuli, as this method gives lower variability in reaction time (RT) and shorter latency.

**Aims:**

This study aimed to assess performance on vibrotactile simple and choice RT tasks between hazardous and non-hazardous drinkers.

**Methods:**

Participants (*n* = 86) completed vibrotactile tasks and alcohol, mood and subjective function (Executive Function Index (EFI)) questionnaires. Multivariate analyses of covariance were performed on average RT scores, and on EFI scores, to investigate function, and a bivariate correlation assessed the relationships between subjective and objective measures.

**Results:**

Hazardous drinkers exhibited significantly faster choice RT. With regard to subjective executive function, Strategic Planning and Impulse Control were significantly better in non-hazardous drinkers. Finally, Organisation and Impulse Control both significantly positively correlated with choice and simple RT, indicating that as subjective function improved, RT increased (a decline in performance).

**Conclusions:**

These results are considered in the context of the premature ageing hypothesis, impulsivity and the impact of alcohol use on various neurotransmitter systems. Furthermore, the poorer subjective function in young hazardous drinkers indicates a possible metacognitive deficit, increased effort or issues with vibrotactile perception as a cognitive function assessment in this group.

## Introduction

Around one-third of the population drink alcohol globally (Griswold et al., 2018), with alcohol named as a leading risk factor for disease burden (Rehm et al., 2003). Evidence suggests that Heavy Episodic Drinking (HED or binge drinking), even at non-clinical levels, is associated with Executive Cognitive Function (ECF) deficits that can affect daily function (Houston et al., 2014; Montgomery et al., 2012). A recent meta-analysis found that young people with HED were significantly impaired relative to controls in ECF (inhibitory control, decision-making) (Lees et al., 2019). Similar results have been observed across broader age ranges of HED adults with impairments in tasks assessing the ECFs response inhibition and cognitive flexibility after controlling for age and gender effects (Houston et al., 2014); increased Stroop RT and decreased accuracy associated with HED with corresponding decreases in brain activity in regions mediating these functions have also been observed (Affan et al., 2018). Carbia et al. (2018)also highlight the effects of HED on response inhibition and to a lesser extent attentional switching and memory updating in their review. While objective assessments of ECF are important in identifying component processes of ECF that may be affected by HED, self-reported problems with ECF function provide an interesting insight into subjective cognitive state which may be more indicative of the effects of HED on cognitive effort required in performing these ECFs in real-world settings. In line with this, heavy drinking has also been shown to affect subjective ratings of ECF, with hazardous drinkers reporting subjectively worse Organisation, Strategic Planning and Impulse Control than non-hazardous drinkers (Powell et al., 2021a) and greater dysexecutive function (Houston et al., 2014).

Processing speed is a task-independent construct that underpins more complex abilities including the ECF outlined above (Fry and Hale, 2000), and determines the efficiency at which cues are interpreted and a task-appropriate response is selected (Fisk and Warr, 1996; Gordon et al., 2018). Processing speed can be thought of as a general construct, but can be divided further into simple psychomotor speed such as the time taken to complete a rapid motor movement, for example, in box completion, horizontal line marking and digit copying, and higher order ‘perceptual’ tasks requiring executive control alongside motor control, for example, colour naming and addition/subtraction tasks (Cepeda et al., 2013). A variety of tasks are often used for ‘reaction time’ (RT), which can be ‘simple’ (one stimulus and one response type) or ‘choice’ (requiring more executive control, usually multiple possible stimuli each requiring a different response) RT (Cepeda et al., 2013). Processing speed is impaired by acute administration of alcohol (Maylor and Rabbitt, 1993; Tzambazis and Stough, 2000), as well as alcohol hangover (Grange et al., 2016). People diagnosed with alcohol use disorders (AUD) also show RT impairments (Crowe, 2019; Stavro et al., 2012).

Hazardous alcohol use has been defined as a pattern of alcohol use that increases risk of harm (World Health Organization, 2019). The relationship between hazardous use and processing speed is unclear. Some studies have shown no difference between hazardous and non-hazardous drinkers. For example, studies using Digit Symbol Substitution and pattern comparison tasks (requiring identification and copying of symbols into a matrix over a set time period) have found no difference in the number of correct substitutions made between HED and controls (Affan et al., 2018; Winward et al. 2014a, 2014b) in addition to absence of effects of age and age × drinking level interactions (Woods et al., 2016). Similarly, tasks requiring letter or number sequencing like the Trail Making Test A (TMT-A) and Delis–Kaplan Executive Function System letter/number sequencing have demonstrated no HED-related differences in the overall time to complete (Winward et al., 2014a, 2014b; Nguyen-Louie et al., 2015), with one study demonstrating that heavier drinkers aged 70 years showed no effects of binge drinking (ranging from 0 to 3+ drinks daily) on TMT-A, and that performance did not decline over the 7 years from time 1 (age 70 years) to time 2 (age 77 years) (Hogenkamp et al., 2014). Congruent Stroop RT has been shown to be comparable between moderate drinkers and HED on a spatial Stroop task (Kashfi et al., 2017). Rodgers et al. (2005) have also reported that light drinkers were superior to abstainers and occasional drinkers in a simple and choice RT task composite score (requiring pressing a response box when a specified light appeared), though hazardous and harmful drinkers did not differ significantly from any of the other groups.

However, using a similar paradigm with vibrotactile presentation of stimuli, drinking level-related differences were observed (Nguyen et al., 2013). There is also evidence that heavier non-dependent drinkers have faster processing speed than their lighter drinking counterparts. For example, Townshend and Duka (2005) demonstrated that binge drinkers were faster in eight-pattern matching to sample choice RT, with no increase in errors indicating that this was not due to a speed-accuracy trade-off. In a longitudinal study, Zanjani et al. (2013) utilised a task requiring finding and matching of figures, finding that overall males showed consistent decline across drinking status (abstainer, moderate drinker, at-risk drinker) while female abstainers showed the greatest decline relative to moderate and at-risk drinkers. This suggests that gender might be an important factor in alcohol-related changes in processing speed. These effects of heavier drinking on processing speed were supported in a recent systematic review including 18 studies assessing processing speed in HED, where HED was found to be associated with significantly faster processing speed in the meta-analysis (Lees et al., 2019). In addition, Piumatti et al. (2018) conducted a longitudinal analysis and found that RT was faster (improved) with every 1 g/day of alcohol but slowed as this increased beyond 10 g/day, with increasing age also identified as a factor in cognitive decline.

While the evidence above suggests little negative effect of heavy alcohol consumption on processing speed, some studies have identified processing speed deficits in heavy drinkers. For example, using the Paced Auditory Serial Addition Task requiring addition of pairs of two-digit numbers at different presentation speeds, people with HED were found to make fewer correct responses at faster presentation rates of 1.2 and 1.6 s (Hartley et al., 2004). Moreover, in a study that controlled for effects of age, sex, physical activity, age of onset of HED and other demographic variables, performance in TMT-A was found to be impaired in HED; there was also a significant effect in females only when stratifying the, sample indicating female HED, but not male HED, performed worse on TMT-A (Salas-Gomez et al., 2016). This was supported by Houston et al. (2014) who found that heavier alcohol consumption was associated with slower TMT-A completion. It is clear from the preceding two paragraphs that there are mixed findings regarding the effects of heavy drinking on processing speed and RT, and that gender, age and classification of drinking status could be potential confounds. For example, methods used to classify drinking behaviours (e.g. interview vs questionnaire, using frequency/quantity of consumption vs broader elements such as grouping into hazardous/non-hazardous vs assessing alcohol use as a continuum, and in studying hazardous drinkers generally vs specific consumption patterns, e.g. HED) varied between individual studies and could result in differential classification of a participant as at risk/hazardous or not.

In addition, the method of processing speed assessment, and the response modality could also affect the results. There were a range of tasks used in previous research that include pencil and paper, manual responding to visual or auditory presentation and manual responding to vibrotactile presentation. Cognitive functions are often objectively assessed using tasks that involve stimulus perception, for example, a RT assessment may rely on the pressing of a button upon seeing or hearing certain stimuli. Moreover, previous research also suggests that impairments may be domain specific, with Woods et al. (2016) finding no difference for perceptual speed, but an impairment in psychomotor speed. Consequently, modality of presentation and response may impact results. Previous research using inhibitory control tasks has identified that inhibition assessed using an auditory Go/No-go task is more consistent in finding impairment when alcohol is administered than when visual stimuli are used (Christiansen et al., 2013; Guillot et al., 2010). Vibrotactile perception, the perception of vibration through touch, can be assessed via tasks that stimulate the fingertips and record responses (Holden et al., 2012), and may be a useful method of assessing cognitive functions for a number of reasons. Firstly, the organisation of the somatosensory system is somatotopic (adjacent regions of the body represented adjacently), and is therefore ideal for inducing cortico–cortical interactions in adjacent or near-adjacent cortical regions (Nelson and Chen, 2008). Secondly, compared to auditory or visual input, it is also easier to limit competing same-sense distractions (Holden et al., 2020; Tommerdahl et al., 2016). With regard to RT assessment specifically, noise can be added by computer systems, core processors, screen refresh rates and other hardware/software processing latencies (Holden et al., 2020). Holden et al. (2019) suggest that tactile stimulation using dedicated hardware is the most accurate method for RT assessment compared to visual stimuli with various response methods, and the one with the least RT variability. To date, most studies have used visual presentation and manual responding (Hogenkamp et al., 2014; Houston et al., 2014; Nguyen-Louie et al., 2015; Winward et al., 2014a, 2004b; Woods et al., 2016; Zanjani et al., 2013). Two studies used dedicated hardware, with Rodgers et al. (2005) using a ‘box’ that displayed lights and had response buttons, and Nguyen et al. (2013) using the dedicated vibrotactile device mentioned previously. These methodological variations could account for some of the variability in findings.

In an earlier study, we used vibrotactile presentation with response via computer mouse to identify alcohol-related changes in processing speed during early residential detox in individuals with an AUD (Powell et al., 2021b). This approach also identified differences in the ability to discriminate between different amplitudes in heavy and light drinkers (Nguyen et al., 2013) and in young (aged 18–26 years) drinkers, and therefore appears sensitive to alcohol-related cognitive changes. The current study aimed to assess simple and choice vibrotactile perceptual RT and subjective ECF between hazardous and non-hazardous drinkers. We hypothesised that (1) hazardous drinkers would have slower RTs than non-hazardous drinkers, (2) hazardous drinkers would report poorer subjective ECF than non-hazardous drinkers and (3) there would be a negative correlation between objective and subjective measures, with slower RT scores (worse performance) correlating with poorer subjective function.

## Methods

### Design

A between-groups cross-sectional design assessed cognitive function via vibrotactile perception tasks and subjectively rated questionnaires between hazardous and non-hazardous drinkers. The independent variable was alcohol use, with two levels: non-hazardous and hazardous (Alcohol Use Disorders Identification Test (AUDIT; Saunders et al., 1993); ⩾8 categorised as hazardous drinking; World Health Organization, 2001). The dependent variables were simple RT, RT variability, choice RT and RT Fatigue and subscales of the Executive Function Index (EFI; Spinella, 2005). Gender, age and mood state were covariates in all main analyses.

### Participants

Potential participants self-identified as eligible if they were aged 18+ and were fluent in English. Exclusion criteria which could affect RT were history of alcohol or substance use disorder, learning disabilities, neurological impairment, pregnancy, use of cocaine within the last month or a condition impacting sensation in dominant hand. A total of 90 individuals took part. Four participants were removed from the main analyses.^1^ Therefore, the study comprised of 86 participants. All individuals lived in the United Kingdom and were recruited from the Northwest of England. Participants were categorised into hazardous (*n* = 36) and non-hazardous drinkers (*n* = 50) using AUDIT score (⩾8 classed as hazardous drinking). Age was significantly higher in the non-hazardous group *t*(83.65) = 2.621, *p* = 0.010 (see Table 1 for participant characteristics).

**Table 1.** Characteristics of participants.

Measure	Non-hazardous (*n* = 50)				Hazardous (*n* = 36)			
	Min	Max	*M*	SD	Min	Max	*M*	SD
Age	18	80	37.40	18.83	18	70	28.00	14.41
AUDIT total	0	6	3.44	1.96	8	22	12.06	3.76
HADS anxiety	0	17	6.78	4.07	3	15	8.61	3.50
HADS depression	0	13	3.02	2.63	0	10	3.08	2.27
*Gender*	Count		Percentage		Count		Percentage	
Female	36		72.0		21		58.3	
Male	14		28.0		15		41.7	
*Educational level*								
Levels 1 – 5 (secondary school – Cert/HNC/HND or equivalent)	19		38.0		21		58.3	
Level 6 (BSc, BA or equivalent)	10		20.0		4		11.1	
Levels 7 and 8 (MSc, MA, doctoral or equivalent)	16		32.0		6		16.7	
Trade, technical or vocational training (level unknown)	4		8.0		5		13.9	
*Employment status*								
Full-time work	10		20.0		8		22.2	
Part-time work	13		26.0		14		38.9	
Student	8		16.0		5		13.9	
Retired	7		14.0		2		5.6	
Unemployed	12		24.0		7		19.4	
*Mental health disorders*								
None	43		86.0		30		83.3	
Reported mental health condition (e.g. anxiety, personality, eating, neurodevelopmental)	7		14.0		6		16.7	
Cognition impacting medication*								
No medication	34		68.0		27		75.0	
Medication which could impact cognition (including contraceptive pill, antidepressants, PPI or H1/H2 antagonist)	14		28		8		22.2	
Other medication not affecting cognition	2		4.0		1		2.8	

HNC: Higher National Certificate; HND: Higher National Diploma; NSAID: non-steroidal anti-inflammatory drugs; PPI: proton pump inhibitors.

* No participants took antiparkinsonian, antibiotic, antipsychotic, pain relief (opioid or NSAID), anticonvulsant, anxiolytic, alcohol-related or high-dose vitamin medications.

### Materials

#### Demographics

Participants answered questions on age, gender, employment status, housing status, education level, mental health diagnoses, medication and country of residence.

#### Subjective executive function

The EFI (Spinella, 2005) is a 27-item five-point (1–5) Likert-type scale assessing five ECF elements derived via factor analysis; Strategic Planning, Motivational Drive, Impulse Control, Organisation and Empathy. Scoring involves summing relevant items (some reversely), and higher total and subscale scores indicate better function.

Scores on the EFI reflect the integrity of prefrontal cognitive abilities, and link well to the factor structure (Spinella, 2005); items group into a three-factor model; with Organisation and Strategic Planning as the first factor, Impulse Control and Empathy and the second factor and Motivational Drive as the third factor. These relate to the model of functional organisation of dorsolateral, orbitofrontal and prefrontal medial circuits (Cummings, 1993; Miller and Cummings, 2017). EFI had a Cronbach’s α total of 0.82 in initial development, an acceptable internal consistency, ranging between 0.69 and 0.76 for the five subscales (Spinella, 2005). In our study, Cronbach’s α totalled 0.80, and ranged from 0.78 to 0.80 across the items. It was lower for the subscales, which were as follows: Motivational Drive = 0.42, Impulse Control = 0.49, Strategic Planning = 0.67, Organisation = 0.74, Empathy = 0.78.

#### Mood state

The Hospital Anxiety and Depression scale (HADS; Zigmond and Snaith, 1983) is a 14-item Likert-type scaled questionnaire used to assess the state of anxiety and depression. In this study, Cronbach’s α totalled 0.80, and ranged from 0.77 to 0.80 across the items. For the subscales it was 0.77 (anxiety) and 0.66 (depression).

*Alcohol use* was assessed using the AUDIT (Saunders et al., 1993), a 10-item, five-point (0–4) Likert-type scaled questionnaire used to indicate hazardous/harmful drinking, via a cut-off score of 8+ (World Health Organization, 2001); in the present study, we did not utilise an upper limit for dependent drinking. AUDIT is validated within the general population (Aalto et al., 2009), and primary health care in six countries (World Health Organization, 2001), and is reliable (Donovan et al., 2006; Fiellin et al., 2000). Currently, Cronbach’s α totalled 0.80 and ranged from 0.74 to 0.81 across the items (though item 6, ‘How often during the last year have you needed a drink first thing in the morning to get yourself going after a heavy drinking session?’ was removed from this internal consistency assessment due to there being no variance as every participant scored 0, ‘Never’).

#### Reaction time

This was assessed using dedicated hardware with an inbuilt microprocessor (the Brain Gauge Pro), which is the same size/shape as a computer mouse. A customised test battery was used to target prefrontal function, with two cylinders (5 mm diameter) delivering vibrotactile stimulation to the middle and index finger of the dominant hand. The device software provides participants with instructions on the computer screen and consists of a series of practice trials, and 10 successive trials, which are separated by a randomised intertrial interval of 2–7 s (Kim et al., 2020; Zhang et al., 2011). All participants in the present study were able to proceed past the practice trials to the main tasks. Participants completed both simple and choice RT as detailed in the procedure section. In addition to the simple and choice RT scores a *RT variability* score (the standard deviation of the 10 trials) and a *Fatigue* score (comparing the first and last tasks) are also generated. Averaged scores of simple RT, RT variability and choice RT were used in all analyses (milliseconds), as was the composite score of Fatigue.

### Procedure

Potential participants were recruited using opportunity sampling via various methods. Student participants were recruited via an internal recruitment database, posters in university buildings, Listserv emails and the Liverpool John Moores University (LJMU) research participation website. Members of the public were recruited via social media adverts (Twitter) and the LJMU Psychology Research Participation Panel. Recruited participants were invited to LJMU for an individual testing session in a psychology laboratory. After giving informed consent, participants completed the vibrotactile tasks (simple, choice and then a repetition of simple to create the Fatigue score). For simple RT, participants were instructed to press the opposing tip (index finger) as soon as they felt a tap (25 Hz, 300 μm, 40 ms) on their middle finger. For choice RT participants were instructed to press the opposing tip as soon as they feel a vibration to the other finger. In this condition, either index or middle finger may be tapped each time, so responding involves choice. Participants first completed a series of practice trials for which they must correctly respond three consecutive times to proceed, and 10 successive trials, which are separated by a randomised intertrial interval of 2–7 s. After completion of the RT tasks, the questionnaires were completed in a counterbalanced fashion. Overall, the testing session lasted between 45 and 60 min per participant and participants were given a debrief sheet explaining the purpose of the study with information about where they can seek help for their/others’ drinking problems if they are concerned, and given a £10 shopping voucher as a thank you for their participation. The study was approved by LJMU Research Ethics Committee (19LJMUSPONSOR0037).

### Statistical analyses

Analyses were conducted using SPSS v28 (IBM Corp., Armonk, New York, USA). To assess differences in mood state between the groups, we used multivariate analysis of variance (MANOVA) with drinking level (hazardous vs non-hazardous) as the between-groups independent variable and HADS anxiety and depression as the dependent variables. Shapiro–Wilk tests using a Bonferroni correction indicated normality of mood state across drinking level was violated for two out of four tests. Due to there being no non-parametric MANOVA equivalent, and due to MANOVA being robust regarding normality violations, this analysis was considered the most appropriate. Two multivariate analysis of covariance (MANCOVA) analyses were performed on average RT scores, and on EFI scores, using drinking level (non-hazardous and hazardous) as the between-groups independent variable. In both analyses, mood state, age and gender were included as covariates, due to their associations with both alcohol use/consequences (Novier et al., 2015; Tovmasyan et al., 2022; White, 2020) and ECF (Best and Miller, 2010; Ferguson et al., 2021; Grissom and Reyes, 2019; Mitchell and Phillips, 2007; Zaninotto et al., 2018). MANCOVA assumptions were assessed, linearity and residual normality were acceptable. For the RT MANCOVA, Box’s test was violated (*p* = 0.01) so Pillai’s Trace statistics are reported. Homogeneity of regression slopes were achieved in all cases except drinking level × gender (*p* = 0.05). Therefore, this violation indicates that a moderator approach would be more appropriate, so the drinking level × gender interaction term is subsequently included in the model. We also created age-related drinking groups and repeated the RT MANCOVA with age-related drinking level (four levels: ‘older’ (30+ years) hazardous; older non-hazardous; ‘younger’ (18–29 years) hazardous; younger non-hazardous) as the between-groups independent variable, average RT scores as the dependent variables and gender and mood state as covariates to assess the effects of age-related drinking level on RT measures. Finally, to investigate relationships between subjective and objective function, a bivariate correlation was conducted.

## Results

Descriptive statistics for mood state, subjective ECF and RT in hazardous and non-hazardous drinkers, and RT in age-grouped drinking levels are displayed in Table 2.

**Table 2.** Descriptive statistics for mood, RT and EFI for hazardous and non-hazardous drinkers.

	Non-hazardous				Hazardous			
	*M*		SE		*M*		SE	
HADS anxiety	6.78*		0.54		8.61		0.64	
HADS depression	3.02		0.35		3.08		0.41	
*MANCOVA adjusted RT scores*								
Simple RT	310.97		9.46		301.01		11.27	
RT variability	28.78		2.89		28.78		3.44	
Choice RT	462.07*		11.84		442.64		14.10	
Fatigue	−9.04		9.39		−12.83		11.19	
*MANCOVA adjusted EFI mean scores*								
Motivational Drive	15.36		0.33		14.49		0.39	
Organisation	16.97		0.43		15.88		0.51	
Strategic Planning	26.75***		0.54		23.48		0.63	
Impulse Control	17.40***		0.32		14.93		0.38	
Empathy	25.70		0.44		26.11		0.52	
	Younger non-hazardous		Younger hazardous		Older non-hazardous		Older hazardous	
*MANCOVA adjusted RT scores for age-grouped drinking levels*								
Simple RT	295.06	14.96	267.19****	14.27	345.73**	14.88	357.18*	25.96
RT variability	22.19	4.26	26.00	4.06	38.84	4.26	26.59	7.39
Choice RT	421.47**	18.99	401.28****	18.11	520.61****	18.88	489.95	32.94
Fatigue	−1.97	13.15	1.64	12.55	−22.27	13.08	−43.85	22.82

Differences significant at: *p < 0.05. **p < 0.01. ***p < 0.001. ****p < 0.0001.

RT: reaction time; EFI: Executive Function Index; AUDIT: Alcohol Use Disorders Identification test; HADS: Hospital Anxiety and Depression scale; SE: standard error; MANCOVA: multivariate analysis of covariance.

Inspection of Table 2 shows that while self-reported depression state scores were comparable between the groups, the hazardous drinking group had higher mean scores for anxiety state indicating higher subjective levels of anxiety. Using MANOVA, the multivariate main effect of drinking level on mood approached significance, *F*(2,83) = 2.86, *p* = 0.06, with univariate analyses demonstrating that anxiety, *F*(1,84) = 4.75, *p* = 0.03, but not depression, *F*(1,84) = 0.01, *p* = 0.91, differed significantly between the groups.

### Reaction time

Table 2 shows that there was little difference between the groups in covariate adjusted means for simple RT, RT variability and Fatigue. There were no significant differences in percentage correct on choice RT between hazardous (93.33%) and non-hazardous (93.80%) drinkers, *F*(1,83) = 0.06, *p* = 0.81]. However, the hazardous drinkers had lower scores for choice RT indicating that they were faster (better) than the non-hazardous drinkers. We used MANCOVA to assess between-group differences in RT measures; for brevity, only multivariate effects are reported in full below (see Table 3 for full MANCOVA statistics). There was a significant multivariate main effect of drinking level on overall RT performance, *F*(4,76) = 2.80 *p* = 0.03, η_p_^2^ = 0.13. Age, *F*(4,76) = 14.56, *p* < 0.001, η_p_^2^ = 0.434, and gender, *F*(4,76) = 3.09, *p* = 0.02, η_p_^2^ = 0.14, were also significant as covariates, as was the gender × drinking level interaction, *F*(4,76) = 2.70, *p* = 0.04, η_p_^2^ = 0.12. State depression, *F*(4,76) = 0.19, *p* = 0.12, and state anxiety, *F*(4,76) = 0.19, *p* = 0.12, were not significant as covariates. Table 3 reveals that age (RT, RT variability, choice RT) and gender (RT variability, choice RT) were both significant covariates for differing RT scores in the MANOVA, while the effects of drinking level on choice RT was the only significant difference after controlling for the effects of age, gender and state mood.

**Table 3.** MANCOVA between subject effects for (i) drinking level on RT controlling for mood state, age and gender with a gender × drinking level interaction term and (ii) age-related drinking level on RT controlling for gender and mood state.

Effects		(i) Drinking level (hazardous vs non-hazardous)			(ii) Age-related drinking group		
		*F*	*p*	η_p_^2^	*F*	*p*	η_p_^2^
Drinking level × gender, *F*(1,79)	RT	0.08	0.78	0.00	–	–	–
	RT variability	0.09	0.76	0.00	–	–	–
	Choice RT	4.69	0.03	0.06	–	–	–
	Fatigue	0.04	0.84	0.00	–	–	–
HADS anxiety, *F*(1,79)	RT	0.09	0.77	0.00	0.05	0.83	0.01
	RT variability	0.37	0.54	0.00	0.34	0.56	0.01
	Choice RT	1.15	0.29	0.01	0.68	0.41	0.01
	Fatigue	1.66	0.20	0.02	2.02	0.16	0.03
HADS depression, *F*(1,79)	RT	0.61	0.44	0.01	1.72	0.19	0.02
	RT variability	3.81	0.05	0.05	3.21	0.08	0.04
	Choice RT	1.17	0.28	0.01	1.93	0.17	0.02
	Fatigue	0.27	0.61	0.00	0.02	0.89	0.01
Age, *F*(1,79)	RT	39.68	0.01	0.33	–	–	–
	RT variability	16.53	0.01	0.17	–	–	–
	Choice RT	43.88	0.01	0.36	–	–	–
	Fatigue	3.62	0.06	0.04	–	–	–
Gender, *F*(1,79)	RT	3.68	0.06	0.04	2.94	0.09	0.04
	RT variability	4.25	0.04	0.05	4.52	0.04	0.05
	Choice RT	7.48	0.01	0.09	5.86	0.02	0.07
	Fatigue	3.06	0.08	0.04	3.11	0.08	0.04
Drinking level, *F*(1,79), or age-related drinking level, *F*(3,79)	RT	0.01	0.94	0.00	6.04	0.001	0.19
	RT variability	0.08	0.77	0.00	2.77	0.05	0.10
	Choice RT	5.61	0.02	0.07	7.91	0.001	0.23
	Fatigue	0.07	0.79	0.00	1.41	0.25	0.05

MANCOVA: multivariate analysis of covariance; RT: reaction time; HADS: Hospital Anxiety and Depression scale.

Due to the significant covariate effect of age in all analyses, we categorised participants as ‘older’ (30+ years) hazardous (*n* = 8) and non-hazardous (*n* = 25) drinkers and ‘younger’ (18–29 years) hazardous (*n* = 28) and non-hazardous (*n* = 24) drinkers, and repeated MANCOVA. The mean scores for these groups in Table 2 demonstrate that the two younger groups have lower (faster) RT scores than the older groups, and that the younger hazardous drinkers are faster than the other groups. There was a significant multivariate main effect of age-related drinking group, *F*(12,234) = 2.77, *p* < 0.001, η_p_^2^ = 0.12, and significant covariate effects of gender, *F*(4,76) = 2.79, *p* = 0.03, η_p_^2^ = 0.13 and depression state, *F*(4,76) = 2.68, *p* = 0.04, η_p_^2^ = 0.12, but not anxiety, *F*(4,76) = 1.87, *p* = 0.12. Pairwise comparisons (Table 4) indicated that young hazardous drinkers performed better than both older groups on simple RT; non-hazardous older drinkers had significantly worse RT variability than non-hazardous younger drinkers; and non-hazardous older drinkers performed worse than both young groups on choice RT.

**Table 4.** Mean differences in pairwise comparisons in MANCOVA of age-related drinking groups.

		Hazardous older	Non-hazardous younger	Non-hazardous older
RT	Hazardous younger	−89.99*	−27.88	−78.54*
	Hazardous older		62.11	11.45
	Non-hazardous younger			−50.67
RT variability	Hazardous younger	−0.58	3.91	−12.84
	Hazardous older		4.40	−12.26
	Non-hazardous younger			−16.65*
Choice RT	Hazardous younger	−88.67	−20.18	119.32*
	Hazardous older		68.49	−30.65
	Non-hazardous younger			−99.14*
Fatigue	Hazardous younger	45.48	3.61	23.90
	Hazardous older		−41.88	−21.58
	Non-hazardous younger			20.30

MANCOVA: multivariate analysis of covariance; RT: reaction time.

* Mean difference significant at *p* < 0.01 after Bonferroni correction.

### Subjective executive function

Table 2 displays the MANCOVA adjusted means for the EFI subscales, indicating that for all subscales except Empathy, non-hazardous drinkers score higher (better subjective ECF). MANCOVA found significant covariate effects of age, *F*(5,75) = 5.94, *p* < 0.001, η_p_^2^ = 0.28, depression state, *F*(5,75) = 6.45, *p* < 0.001, η_p_^2^ = 0.30 and anxiety, *F*(5,75) = 6.34, *p* < 0.001, η_p_^2^ = 0.30, but not of gender, *F*(5,75) = 0.75, *p* = 0.60. After covariates were controlled for, there was a significant multivariate main effect of drinking group on subjective ECF, *F*(5,75) = 7.56, *p* < 0.001, η_p_^2^ = 0.34. Follow-up univariate ANCOVAs found that while non-hazardous drinkers reported better subjective ECF on all measures (except for Empathy), this difference was only significant for Strategic Planning, *F*(1,79) = 14.38, *p* < 0.001, η_p_^2^ = 0.154 and Impulse Control, *F*(1,79) = 22.81, *p* < 0.001, η_p_^2^ = 0.224. There were no significant differences between hazardous and non-hazardous drinkers for Motivational Drive, Organisation or Empathy, *p* = 0.11, 0.12 and 0.57, respectively).

### Subjective and objective Function

To assess the relationships between subjective and objective function, bivariate correlations (Kendall’s τ) were run on average RT scores and EFI subscale scores (see Table 5). There were significant positive associations between Organisation and simple, τ_b_ = 0.20, *p* = 0.01, and between Impulse Control and simple, τ_b_ = 0.25, *p* = 0.001, and choice RT, τ_b_ = 0.25, *p* = 0.001. This suggests that as subjective function improved, RT performance worsened (response latency increased).

**Table 5.** Kendall’s τ correlation matrix for RT and subjective executive function.

	Simple RT	RT variability	Choice RT	Fatigue	EFI-MD	EFI-ORG	EFI-SP	EFI-IC
RT variability	0.393*	–						
Choice RT	0.451*	0.280*	–					
Fatigue	−0.173	−0.082	0.120	–				
Motivational Drive	0.091	0.069	0.077	0.043	–			
Organisation	0.198*	0.093	0.161	−0.002	0.214*	–		
Strategic Planning	0.084	0.041	0.116	0.125	0.255*	0.249*	–	
Impulse Control	0.252*	0.103	0.251*	0.032	0.218*	0.452*	0.200	–
Empathy	−0.030	−0.115	−0.019	−0.028	0.113	0.042	0.161	0.398

RT: reaction time; EFI: Executive Function Index; MD: Motivational Drive; ORG: Organisation; SP: Strategic Planning; IC: Impulse Control.

* *p* ⩽ 0.01 (two-tailed).

## Discussion

This study assessed hazardous drinking-related differences in vibrotactile simple and choice RT. In contrast to hypothesis 1, hazardous drinkers were faster than non-hazardous drinkers at choice RT, though they reported poorer subjective EF. There was a positive correlation between objective and subjective measures, slower simple or choice RT scores (worse performance) correlated with better self-reported ECF on certain EFI subscales (Organisation and simple RT, and Impulse Control and simple and choice RT). After controlling for covariates, hazardous drinking was associated with faster choice RT, but not with simple RT, RT variability or Fatigue. This suggests that hazardous drinkers, in this study, were better at responding quickly on the more executive-oriented task, but that this advantage did not extend to simple RT, the variability between simple RT trials (an indicator of attention) or the Fatigue score.

Research with clinical populations of people with AUD consistently shows impaired processing speed (Crowe, 2019; Stavro et al., 2012). It has been assumed that hazardous drinking can be considered a precursor stage to developing an AUD, and therefore that many of the impairments observed at the dependent stage would be seen in hazardous drinkers, albeit to a lesser extent (Lees et al., 2019). However, the current results challenge this assumption, and are more consistent with other studies showing faster RT in hazardous drinkers (Bø et al., 2016; Hartley et al., 2004; Kashfi et al., 2017; Lees et al., 2019; Mazumder et al., 2021; Townshend and Duka, 2005; Zanjani et al., 2013), and those that show no relationship (Affan et al., 2018; Cohen-Gilbert et al., 2017; Hogenkamp et al., 2014; Nguyen et al., 2013; Rodgers et al., 2005; Winward et al., 2014a, 2014b; Woods et al., 2016). This finding is of interest and suggests that perhaps hazardous drinkers require less time to make a choice in a choice RT task than non-hazardous drinkers (as proposed by Townshend and Duka, 2005). There are a number of possible tentative explanations for this. Firstly, in animal models, acute alcohol administration reduces longer RTs on the five Choice Serial Reaction Time task, with longer RT emerging during abstinence, and peaking 30 days after last acute administration (Wright et al., 2013). Consequently, it is possible that in the present study, the hazardous drinkers were faster due to recent heavier alcohol use, and that slower RT might have become apparent under longer periods of abstinence. Higher levels of gamma-aminobutyric acid (GABA) due to recent heavy alcohol consumption could lend support to this explanation. GABA increases cortical inhibition and thus higher GABA may be beneficial for tasks involving response selection, as it limits neuronal noise, enabling selective neural activity (de la Vega et al., 2014; Munakata et al., 2011; Snyder et al., 2010).

Secondly, while in the present study it is unlikely that the increased RT reflects a speed-accuracy trade-off as there were no significant between-group differences in percentage correct in choice RT, it is possible that the choice RT task was too simple to elicit errors, with only two possible choices. Other studies that have found a speed-accuracy trade-off have used more complex choice RT tasks, or those that require adaptive learning after responding, for example, Bø et al.’s (2016) adaptive Go/No-go where people with HED were faster but failed to adapt to incorrect responses in line with controls. Such speed-accuracy trade-offs are often seen in ECF tasks measuring response inhibition, though tasks assessing this ECF do not solely measure response inhibition, and include elements of processing speed; such as average RT in the Go/No-go task, mean RT in Go trials of the Stop-Signal Task and prosaccade latency in the Antisaccade task (Weiss and Luciana, 2022). As described in the introduction, in one previous study that found faster processing, there was a speed-accuracy trade-off (quicker responses but fewer correct choices), interpreted as indicating an inhibitory control deficit (Kashfi et al., 2017), which may in part explain the initiation of hazardous alcohol use (Blakemore and Robbins, 2012; Gullo and Dawe, 2008). However, in Cohen-Gilbert et al. (2017); Townshend and Duka (2005), several of the studies assessed in Lees et al. (2019), and in the current study, there was no evidence of such a trade-off, even though individuals who responded faster were those who scored lower on the subjective Impulse Control subscale of the EFI. While impulsivity is often viewed negatively, perhaps in some circumstances (particularly those with low capacity for risk) it can lead to favourable outcomes (Gullo and Dawe, 2008). Alternatively, as suggested by Scaife and Duka (2009), the choice RT task may not be complex enough to produce errors in performance at this level of alcohol use, regardless of impulsivity. Another consideration is that young adult drinkers may be faster due to better response monitoring (slowing down following errors, allowing success/failure to guide performance) (Bø et al., 2016), which was not assessed in the current study.

In the age-related drinking group analysis, the participants demonstrating fastest processing speed on simple RT were younger hazardous drinkers, while those with the poorest speed on choice RT were non-hazardous older drinkers. Considered against the ‘premature aging hypothesis’, where AUD in clinical populations may either accelerate ageing of the brain in individuals of any age, or brains of older drinkers with AUD may be more vulnerable to the effects of alcohol (Ellis and Oscar-Berman, 1989; Oscar-Berman and Marinkovic, 2003; Oscar-Berman et al., 2000), this finding in non-clinical hazardous drinkers suggests that the phenomena may not be so clear cut. One study comparing whole-brain contrasts of patients with AUD and controls, provided support for the premature ageing hypothesis, suggesting that increased age increases vulnerability to the cognitive effects of alcohol, and that youth provides protection (Guggenmos et al., 2017). Therefore, considering the current finding that young hazardous drinkers performed better than all older drinkers at simple RT, perhaps the performance difference is preexisting, but alcohol use eventually negates this, just not to the extent of clinical cases of AUD, as hazardous older drinkers were no worse than the other groups. As processing speed is a task-independent construct (Fry and Hale, 2000), it is unlike other functions examined in the literature. Indeed, the findings regarding higher order ECF in hazardous drinkers are more inconsistent in younger drinkers, while older drinkers generally display impairment compared to controls, likely due to a neurocompensatory mechanism of increased cognitive effort/neuronal labour in younger subjects (Gil-Hernandez et al., 2017). Furthermore, some of the processing speed studies previously mentioned found higher brain activation in areas supporting cognitive processes during tasks, which was interpreted as possible neurocompensation (Affan et al., 2018; Kashfi et al., 2017; Pérez-García et al., 2022). The systematic review by Lees et al. (2019) also found greater brain activity during tasks involving attention, inhibition and working memory in HED. It is worth considering whether perhaps an initial processing speed advantage in younger hazardous drinkers could contribute to their ability to perform executive tasks at a comparable level to non-hazardous drinkers, and future research should seek to clarify this.

The finding of poorer subjective function in hazardous drinkers initially appears to contrast with the result of better processing speed. Additionally, the finding of a positive correlation between objective and subjective function is intriguing, as those who were fastest, reported worse day-to-day subjective function. However, given that the strongest relationship was found between Impulse Control and the RT scores, this suggests that slower individuals may have been more prone to thinking before acting. That there was no speed-accuracy trade-off limits this theory, but again, may be due beneficial elements of impulsivity (Gullo and Dawe, 2008), or the relatively easy choice RT task (Scaife and Duka, 2009). Alternatively, this finding may be due to other alcohol effects, such as on metacognition (Le Berre et al., 2017), increased cognitive effort required for tasks (neurocompensation, as described) or methodological issues with vibrotactile perception as an assessment in this cohort. To assess the possibility of metacognition, future studies should compare subjective assessment with validated ECF tasks, alongside processing speed, considering how each of these interrelates. Processing speed in this context should also be assessed using a range of modalities and difficulties, to ensure the previously reported high-RT accuracy of the Brain Gauge compared to other modalities (Holden et al., 2019) is replicable relating to alcohol use, and to further examine accuracy and inhibitory control. Assessment of neural activity during these varied processing speed tasks would also be beneficial.

It is important to note that while the current study found faster processing in hazardous drinkers, particularly in younger hazardous drinkers, the literature is obviously still inconsistent, and the study is not without its limitations. Firstly, while the article used two versions of the RT task, neither were particularly complex, which as mentioned, may have disguised any speed-accuracy disadvantages of quick responding (Scaife and Duka, 2009), indicating that future researchers should use a range of tasks to assess processing speed. Secondly, while it is interesting to speculate about causes for the current findings, this study did not use direct brain measurements relevant to processing speed. Future research could assess hazardous and non-hazardous drinkers using structural methods linked to neural transmission speed, such as those assessing myelination (via recent myelin magnetic resonance imaging techniques (van der Weijden et al., 2021) or indirectly through diffusion tensor imaging (Aung et al., 2013; Song et al., 2002)), or functional methods that assess temporal information about neural processes, such as event-related potentials (ERP). A further limitation of the current study is that it did not assess across patterns of hazardous drinking (e.g. daily drinking vs HED). Maurage et al. (2012) found ERP deficits associated with specific drinking patterns, indicating that researchers should consider how these different patterns affect function. While roughly equal numbers of hazardous (26%) versus non-hazardous (28%) drinkers were tested in the morning versus afternoon testing session, we cannot rule out that some of the effects may have been due to the effects of individual circadian rhythms on cognitive function (Adan, 1993; Valdez, 2019). Future research should seek to assess participants’ circadian preferences (via e.g. the morningness-eveningness questionnaire) and allocate to a testing session as appropriate, and consider how circadian rhythmicity may also confound via influence on alcohol use behaviours (Adan, 2013). Cronbach’s α also revealed varied internal consistency for the subscales of the EFI. These were acceptable in total, across the items, and for the subscales Organisation and Empathy, but were poor for the subscales Motivational Drive, Impulse Control and Strategic Planning. This indicates that any interpretations of the analyses using the EFI must be considered potentially unreliable, particularly those about the subscale Impulse Control. Future research assessing subjective function should consider whether other tools such as the Behaviour Rating Inventory of Executive Function-Adult (Roth et al., 2013) or the Comprehensive Executive Functions Inventory (Naglieri and Goldstein, 2013) would be more appropriate. Finally, the relatively small sample size (particularly in the groups in the age-related drinking group analysis), and lack of a priori power calculation reduces dependability of the findings.

In conclusion, we found that hazardous drinkers were significantly faster at choice RT, and when examined in age-groups, younger hazardous drinkers were fastest at simple RT, while older non-hazardous drinkers were poorest at choice RT. This was discussed in the context of the premature aging hypothesis, impulsivity and neurotransmitters. Furthermore, subjective function was poorer in hazardous drinkers, specifically in young hazardous drinkers, indicating either a possible metacognitive deficit, increased effort or issues with vibrotactile perception assessment in this cohort. Further research should use additional methods to assess RT in hazardous drinking, including assessing neurotransmitter or functional temporal activity during vibrotactile RT, comparing vibrotactile RT with other objective assessments and examining whether these assessments differ across different hazardous drinking patterns.

## Notes

1. One participant was removed from all analyses due to nerve damage in their dominant hand that was not disclosed until testing was complete. Three participants were removed due to initial boxplot outlier inspection revealing that they had invalid scores due to not meeting the choice reaction time response threshold required.
